# Biofilm Formation Reduction by Eugenol and Thymol on Biodegradable Food Packaging Material

**DOI:** 10.3390/foods11010002

**Published:** 2021-12-21

**Authors:** Pavel Pleva, Lucie Bartošová, Daniela Máčalová, Ludmila Zálešáková, Jana Sedlaříková, Magda Janalíková

**Affiliations:** 1 Department of Environmental Protection Engineering, Faculty of Technology, Tomas Bata University in Zlin, 275 Vavreckova, 76001 Zlin, Czech Republic; ppleva@utb.cz (P.P.); l1_bartosova@utb.cz (L.B.); d_macalova@utb.cz (D.M.); 2 Department of Food Technology, Faculty of Technology, Tomas Bata University in Zlin, nam. T. G. Masaryka 5555, 76001 Zlin, Czech Republic; lzalesakova@utb.cz; 3 Department of Fat, Surfactant and Cosmetics Technology, Faculty of Technology, Tomas Bata University in Zlin, 275 Vavreckova, 76001 Zlin, Czech Republic; sedlarikova@utb.cz

**Keywords:** antimicrobial activity, biofilm, biodegradable polymers, food packaging

## Abstract

Biofilm is a structured community of microorganisms adhering to surfaces of various polymeric materials used in food packaging. Microbes in the biofilm may affect food quality. However, the presence of biofilm can ensure biodegradation of discarded packaging. This work aims to evaluate a biofilm formation on the selected biodegradable polymer films: poly (lactic acid) (PLA), poly (butylene adipate-co-terephthalate) (PBAT), and poly (butylene succinate) (PBS) by selected bacterial strains; collection strains of *Escherichia* *coli*, *Staphylococcus* *aureus*; and *Bacillus pumilus*, *Bacillus* *subtilis*, *Bacillus* *tequilensis*, and *Stenotrophomonas* *maltophilia* isolated from dairy products. Three different methods for biofilm evaluation were performed: the Christensen method, 3-(4,5-dimethylthiazol-2-yl)-2,5-diphenyltetrazolium bromide (MTT) assay, and fluorescence microscopy. High biofilm formation was confirmed on the control PBS film, whereas low biofilm formation ability was observed on the PLA polymer sample. Furthermore, the films with incorporated antimicrobial compounds (thymol or eugenol) were also prepared. Antimicrobial activity and also reduction in biofilm formation on enriched polymer films were determined. Therefore, they were all proved to be antimicrobial and effective in reducing biofilm formation. These films can be used to prepare novel active food packaging for the dairy industry to prevent biofilm formation and enhance food quality and safety in the future.

## 1. Introduction

Biofilm is a community of microorganisms attached to a surface and surrounded by an extracellular polymeric matrix. Biofilm provides better living conditions for microorganisms than planktonic form because it maintains the stability of the internal environment, isolates them from the outside, and protects inner cells [1,2,3]. They are more resistant to harmful effects, such as antimicrobial compounds, UV radiation, bacteriophages, antibiotics, and the human immune system. Biofilm formation is a multi-step process starting with a reversible attachment to a surface aided by intermolecular forces and hydrophobicity [4,5]. In the food industry, microorganisms can adhere to abiotic surfaces, form a biofilm, and reduce the shelf life of food products. Furthermore, it may increase the incidence of foodborne illnesses, which is a significant concern for public health and food quality [6,7].

The critical function of food packaging is to protect products from extrinsic factors, especially gases, temperature, and relative humidity [8]. Therefore, active packaging is a system that actively changes the conditions of packaged food and maintains or extends the product quality [9,10]. Food packaging legislation is defined mainly at a national, European, and worldwide level. In the EU, legislative regulations are divided into food packaging, plastic material and articles intended to direct contact with food, and safe use of active and intelligent packaging [8,11,12,13]. Regulation (EC) 450/2009 (European Commission 2009) defines specific rules for active and intelligent materials and articles [9,11]. Regulation (EC) 1935/2004 and Regulation (EC) 10/2011 state that materials and articles, including active and intelligent materials and articles, shall be manufactured in compliance with suitable manufacturing practices (Regulation (EC) No. 2023/2006) [8,12]. Due to the deliberate interaction of active packaging, the migration of substances could pose a food safety problem [9]. Regulation (EC) No. 10/2011 specifies rules for the valuation of the release of low molecular weight substances from plastic in the food packaging and a list of substances of specific threshold limits. The European Food Safety Authority (EFSA) recommends these limits [8,12]. In the United States and Canada, the market requires the Food and Drug Administration (FDA) to approve materials and articles for food contact [14].

Despite possible negative impacts, bacterial biofilms may also have beneficial biodegradable effects [14]. High molecular weight polymers are more difficult to degrade because of their poor solubility and hindered penetration into the cell wall where they are enzymatically degraded. Biodegradable polymers are submitted to biodegradation processes in nature easier than synthetic [14,15]. However, a biofilm on biodegradable polymers may influence the change of mechanical properties, including tensile strength. On the other hand, non-biodegradable polymers, such as polyolefins, are not expected to change the mechanical characteristics [14,16].

With a growing negative view of environmental pollution, biodegradable polymers attract increasing attention, mainly in the fields of packaging materials, agriculture, and medicine [16,17]. Many types of biodegradable polymers can be used in food packaging. They may include conventional polylactide (PLA), polybutylene succinate (PBS), polyamides (PA), poly (butylene adipate-co-terephthalate) (PBAT), polypropylene (PP), and many others [18,19]. They are positively evaluated in the food industry, especially for their diverse mechanical properties, tear resistance, and health safety [20,21]. Chemical and biological additives, such as antioxidants, lubricants, stabilizers, pigments, are often applied to polymeric materials to enhance their properties [22]. Eugenol and thymol, natural phenolic compounds occurring in plant essential oils, could be used for their known antimicrobial properties [23,24]. These added compounds may be rapidly degraded due to their instability and high volatility [25].

Microbial biofilm on packaging material may threaten food safety and quality; what is more, it is also required for their biodegradation after disposal. This study aims to investigate biofilm formation by food isolates on biodegradable polymers used as food packaging and enhance the antibacterial properties of these polymers by enriching them with thymol and eugenol.

## 2. Materials and Methods

### 2.1. Materials and Chemicals

Biodegradable polymers: poly(lactic) acid—PLA (NatureWorks, Minnetonka, MN, USA), poly(butylene adipate-co-terephthalate)—PBAT Ecoflex^®^ (BASF, Ludwigshafen, Germany), and poly(butylene succinate)—PBS G4560 (IRe Chemical Ltd., Seoul, Korea) were tested in this study.

Bacterial strains of *Bacillus tequilensis* R23, *Bacillus subtilis* R25, *Bacillus pumilus* R34, *Stenotrophomonas maltophilia* GK CIP 1/1 were obtained from the Dairy Research Institute in Prague (Czech Republic). These strains were isolated from dairy products. Bacterial strains *Escherichia coli* ATCC 25922 and *Staphylococcus aureus* ATCC 25923 were provided by the Czech Collection of Microorganisms (CCM, Brno, Czech Republic). *Escherichia coli* ATCC 25922 and *Staphylococcus aureus* ATCC 25923 were selected for testing as typical model organisms representing both Gram-negative and Gram-positive bacteria. Isolates from dairy products were chosen as representatives of natural contaminants due to the possibility of future application of tested materials as food packaging. All strains were cultivated in BHI medium (brain heart infusion broth, HiMedia, Mumbai, India). In addition, the culture medium was enriched with 5% sucrose (HiMedia, Mumbai, India) to test biofilm formation. *E. coli* and *S. aureus* strains were cultivated at 37 °C/24 h, other strains were grown at 30 °C/24 h. Mueller Hinton agar (HiMedia Laboratories Pvt. Ltd., Mumbai, India) was used to determine antibacterial activity.

Eugenol and thymol were provided by Sigma-Aldrich (St. Louis, MO, USA). Other used chemicals were obtained from specified companies: chloroform (Penta chemicals, Chrudim, Czech Republic); 96% ethanol (Lach-Ner, Neratovice, Czech Republic); crystal violet (Penta, Prague, Czech Republic); 3-(4,5-dimethylthiazol-2-yl)-2,5-diphenyltetrazolium bromide (MTT) powder (Serva, Heidelberg, Germany); DMSO (Sigma-Aldrich, St. Louis, MO, USA).

### 2.2. Film Preparation

Tested biodegradable polymers (PLA, PBAT, PBS) were prepared in the form of films. The mixture of polymer and chloroform in the concentration of 14% *w*/*v* was homogenized under the continuous stirring (500 rpm, 48 h) at 25 ± 1 °C (except PBS/50 ± 1 °C). The active compounds (eugenol—E, thymol—T) were added into the polymer solution in the final concentration of 3% *w*/*v* and stirred (500 rpm, 1 h) at 25 ± 1 °C. The samples of PLA/E; PLA/T; PBAT/E; PBAT/T; PBS/E; PBS/T were prepared. The solution without active compounds was used as the control. The prepared mixture (10 mL) was poured into Petri dishes (6 cm in diameter) and left to dry in an air-circulated oven (30 °C, 24 h) [26]. A higher concentration of active compounds was chosen to ensure a more extended provision of antimicrobial properties. Tested active compounds in the concentration of 3% *w*/*v* should provide antimicrobial protection for several months for water-based foods, using the result from Narayanan et al. [27].

### 2.3. Antibacterial Activity

Antibacterial activities of PLA, PBS, and PBAT control films and films with eugenol and thymol were tested by the standard agar diffusion test with the following modifications [28]. The films were cut into disks (5 mm in diameter). Before testing, the samples were surface sterilized by UV radiation for 20 min without any sample damage [29]. They were placed on Mueller Hinton agar plates inoculated with 1 mL of 0.5 McF turbid bacterial suspension (*Bacillus tequilensis* R23, *Bacillus subtilis* R25, *Bacillus pumilus* R34, *Stenotrophomonas maltophilia* GK CIP 1/1, *Escherichia coli* ATCC 25922 and *Staphylococcus aureus* ATCC 25923) in the sterile saline solution. *E. coli* and *S. aureus* strains were cultivated at 37 °C/24 h, other strains were grown at 30 °C/24 h. The inhibition zones were evaluated. All experiments were repeated in triplicate.

### 2.4. Biofilm Formation

#### 2.4.1. MTT Assay

The films were cut into squares of 25 mm^2^, placed in a sterile tube with 4.5 mL BHI broth, and inoculated with 50 μL of a 24 h bacterial culture suspension with bacterial turbidity of 1 McF. The cultivation of *E. coli* and *S. aureus* was performed at 37 °C/48 h. For the other strains, it was at 30 °C/48 h with constant stirring on a shaker. The solution was aspirated, and then the samples were rinsed with sterile distilled water to remove the planktonic cells. MTT powder was dissolved in ultrapure water at a 5 mg/mL concentration. The rinsed material was placed into individual wells of a microplate together with 180 μL of BHI broth, and MTT solution was added to the final concentration of 0.5 mg/mL. After the treatment with MTT for 4 h at 30 °C and shaking, the solution was aspirated and replaced with 200 μL of dimethyl sulfoxide (DMSO), which dissolved the formed formazan. Subsequently, 100 μL of formazan solution was placed into each well of a microplate, the absorbance (optical density) at 690 nm was measured, and the background was read at 570 nm using a Tecan Infinite^®^ 200 PRO (Tecan, Mannedorf, Switzerland). The average and standard deviation of the optical density (OD) of negative controls (OD_NC_) were calculated from the measured values. According to Stepanović et al., the cut-off value for positivity (OD_p_) was calculated as the sum of three times the standard deviation and OD_NC_ (OD_p_ = OD_NC_ + 3 × standard deviation OD_NC_) [30]. The resulting biofilm formation was evaluated into 3 categories by limit values:

- Non-biofilm formation (−): OD ≤ OD_P_;
- Weak biofilm formation (+): OD_P_ < OD ≤ 2OD_P_;
- Strong biofilm formation (++): 2OD_P_ < OD.

#### 2.4.2. Christensen Method

The films (25 mm^2^) were placed in a sterile tube with 4.5 mL BHI and inoculated with 50 μL of 1 McF turbid bacterial suspension. After the cultivation (see Section 2.4.1), the material was rinsed thoroughly with sterile distilled water to remove the adhering planktonic cells. Subsequently, 200 μL of 96% ethanol was added for 20 min. The ethanol was then washed and replaced with 200 μL of crystal violet, leaving it to act for 20 min. The stained sample was placed in a test tube with 200 μL of 96% ethanol, which dissolved the bound dye. Next, 100 μL of colored ethanol solution was transferred to each well of a microplate, and then the absorbance at 600 nm was measured using a Tecan Infinite^®^ 200 PRO (Tecan, Männedorf, Switzerland). The limit values for the evaluation of a biofilm formation were determined in the same way as for the MTT assay (see Section 2.4.1).

#### 2.4.3. Fluorescence Microscopy

The samples (25 mm^2^) were washed with sterile saline solution after the cultivation (see Section 2.4.1) and placed on a glass slide. They were dyed by fluorescence dye (SYTO^®^9 and propidium iodide) for 10 s and then covered with a square coverslip. Fluorescence microscopy was performed using a fluorescence microscope Olympus BX53 (Olympus, Tokyo, Japan) equipped with Microscope Digital Camera DP73 (Olympus, Tokyo, Japan) and the cell Sens Standard 1.18 (Olympus, Tokyo, Japan) software. The analysis was performed on a minimum of 20 positions in three replicates. LIVE/DEAD™ BacLight™ Bacterial Viability Kit (Thermo Fisher, Waltham, MA, USA), based on the protocol [31], was executed using slight modifications. SYTO^®^9 dyed plasma membranes of all bacteria, while propidium iodide can color DNA of only dead cells. The excitation/emission maxima for these dyes are 480/500 nm for SYTO^®^9 stain and 490/635 nm for propidium iodide. Thus, bacteria with intact cell membranes stain fluorescent green, whereas bacteria with damaged membranes (dead) stain fluorescent red.

### 2.5. Material Properties

#### 2.5.1. FTIR-ATR Analysis

The chemical composition of prepared films was characterized by Fourier transform infrared spectroscopy (FTIR) on Nicolet 6700 spectrometer (Thermo Fisher Scientific, Waltham, MA, USA) set to ATR mode and fitted with a diamond crystal equipped with OMNIC Paradigm software. Measurement conditions comprised 64 scans at the resolution of 2 cm^−1^ and the range of 4000 to 400 cm^−1^.

#### 2.5.2. Contact Angle Measurement

The wettability of polymer films was measured by the sessile drop method at ambient temperature on a Theta optical tensiometer (Biolin Scientific, Göteborg, Sweden) equipped with OneAttension software. Distilled water of the volume equaling 3 µL was applied as the reference liquid.

### 2.6. Statistical Analysis

The statistical evaluation of the contact angles employed one-way analysis of variance (ANOVA) using Statistica software (version 10, StatSoft, Inc., Tulsa, OK, USA), at the significance level of *p* < 0.05.

## 3. Results

### 3.1. Antibacterial Activity

A method based on the diffusion of active compounds, the disk diffusion method, was used to determine the antimicrobial activity. The inhibition zones were measured, and the values, including the disk diameter (5 mm), are shown in Table 1.

The samples without incorporated active compounds did not inhibit the tested bacteria. In contrast, films enriched with antimicrobial compounds, eugenol, and thymol, have formed the inhibition zones confirming the antimicrobial activity (Table 1). These results corresponded with the published results of eugenol and thymol inhibition effect against bacteria [23,24]. The best inhibitory effect of synthetic polymers PLA, PBS, and PBAT with added phenolic compounds were performed by films enriched with 3% *w*/*v* eugenol. Except for Gram-negative bacteria *E. coli*, all tested isolates from dairy products were inhibited by PLA/E. The samples of PBS/E and PBAT/E performed a complete inhibitory effect against all tested bacteria. The sample of PBS/E showed the largest inhibition zone against *S. aureus*, with the zone reaching the diameter of 17.8 ± 0.5 mm. The polymer films with 3% *w*/*v* thymol presented generally lower antibacterial activities than eugenol films. PLA/T produced the smallest inhibition zones against the tested bacteria with the observed activity only against *B. pumilus*, *S. maltophilia*, and *S. aureus*. The internal structure of the polymer may have an important impact on the release and diffusion of the active compound to the external environment. It may be concluded that incorporating eugenol or thymol into biodegradable polymer films enhances their antimicrobial properties.

### 3.2. Biofilm Formation

Biodegradable polymers are used as an alternative to disposable packaging in the food industry [32]. Bacterial biofilm can grow on these polymers. What is more, it is necessary for their biodegradation [33]. Therefore, it is important to identify a suitable active compound with the optimal concentration not only for extending the shelf life of food but also for prolonged biodegradation. This study examined three methods (MTT assay, Christensen method, and fluorescence microscopy) to monitor biofilm formation on packaging materials.

MTT assay and Christensen method are spectrophotometric methods based on the biofilm staining. In the Christensen method, biofilm is stained with crystal violet. Subsequently, the color is leached with ethanol, corresponding to the strength of a biofilm formation [34]. In contrast, in MTT assay, only the number of living cells in a biofilm is monitored based on their metabolic activity. In MTT assay, living cells reduce yellow MTT to violet formazan. Subsequently, formazan is dissolved in dimethyl sulfoxide (DMSO), and the color of the extract is directly proportional to the number of viable cells [35]. Fluorescence microscopy allows the observation of biofilm viability. Differentiation of living cells from dead cells can be accomplished by staining with propidium iodide, which penetrates the disrupted cytoplasmic membrane of dead cells [36]. Propidium iodide is a red fluorescent nucleic acid that excites at 490 nm and emits 635 nm. SYTO^®^ 9 green-fluorescent nucleic acid, which excites at 480 nm and is emitted at 500 nm, could be used to stain all cells. Using propidium iodide with SYTO^®^ 9 reduces the fluorescence of the SYTO^®^ 9 dye, thereby distinguishing red fluorescent dead cells from green living cells [31].

For both, Christensen method and MTT assay, a biofilm formation was examined by determining the limit values for prepared materials. The limit values were determined from the reference value, i.e., without inoculation with a microorganism and three standard deviations. These values are unique for every single prepared polymer film. The limit value was selected based on a normal (Gaussian) distribution, i.e., the average together with three standard deviations cover almost all probable values (99.7%) [37]. A *p*-value of <0.003 was considered to be statistically significant. The resulting biofilm formation was classified into three categories (non-biofilm formation, weak biofilm formation, and strong biofilm formation). The non-forming biofilm showed an average absorbance value lower than the limit values. Weak biofilm formation achieved values twice the limit value, and above this value, the strains were considered to form a strong biofilm. In the Christensen method, PLA and PBS were stained too much. Strong staining of the surfaces of the tested samples is inappropriate due to the requirement for the subsequent dilution for the samples with a stronger biofilm. It complicates the method and prolongs the time required for the test. Therefore, the Christensen method may be inappropriate for many polymers.

Three methods for the detection of biofilm formation on biodegradable polymer films by *Bacillus tequilensis*, *Bacillus subtilis*, *Bacillus pumilus*, *Stenotrophomonas maltophilia*, *Escherichia coli*, and *Staphylococcus aureus* were compared in order to select the most suitable one (Table 2 for pure materials, Supplementary Materials Table S1 for all tested materials).

As all tested strains are able to form a biofilm, its production was expected on all tested biodegradable polymers. Neither the MTT method nor the Christensen test performed relevant results on PLA. Limoli et al. described that the absence of a detectable biofilm could be caused by the smooth surface of the sample [38]. The results indicate that the MTT method was more suitable than Christensen for PBS, test in a biofilm detection in vitro (Table 2). Nevertheless, the microplate adherence test described by Christensen provided better results on PBAT.

The presence of adherent cells on PLA was confirmed by fluorescence microscopy. MTT method proved the production of a weak biofilm on PBS within all tested strains. On the other hand, only two isolates (*Bacillus pumilus*, *Stenotrophomonas maltophilia*) demonstrated a biofilm using the Christensen method on PBS. On PBAT, a weak biofilm was detected by the Christensen method and fluorescence microscopy. However, no biofilm was detected by MTT assay. These results demonstrate a weak biofilm formation with low metabolic activity on PBAT [35].

When comparing the tested methods, Christensen’s method for detecting a biofilm formation on polymers appeared to be unsuitable due to the strong dyeability of polymers themselves. Another disadvantage is that MTT assay and Christensen method are time-consuming. What might be considered beneficial of these methods is that the samples are not rinsed during this test. A biofilm may be destroyed when rinsed, causing a false negative result [5].

**Figure 1:**
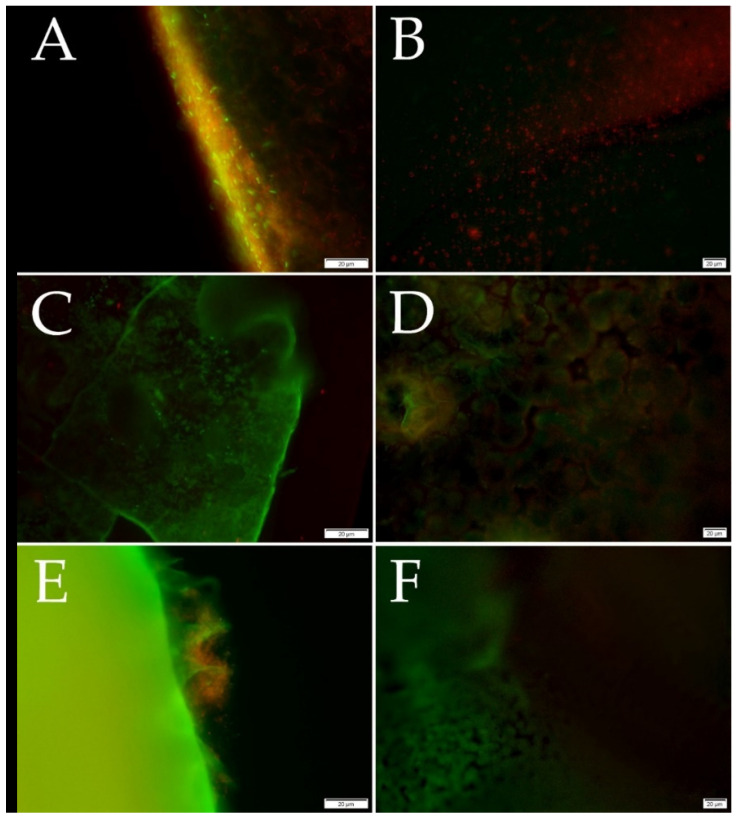
Fluorescence microscopy of LIVE/DEAD bacterial viability assay with *Stenotrophomonas maltophilia*. (**A**): PLA, (**B**): PLA/T, (**C**): PBS, (**D**): PBS/T, (**E**): PBAT, (**F**): PBAT/T. PLA: poly(lactic) acid, PBAT: poly(butylene adipate-co-terephthalate), PBS: poly(butylene succinate), T: 3% *w*/*v* thymol.

Based on the obtained results, it could be concluded that a biofilm on the polymer films was precisely detected using fluorescence microscopy. LIVE/DEAD bacterial viability assays were performed by fluorescence microscopy, as can be seen in Figure 1. Live bacterial cells are displayed green, and dead bacterial cells are red. It seems that an insignificant difference was found between all neat polymers. However, the spherulite-forming PBS does not provide a suitable surface for bacterial adhesion. Thus, the bacteria accumulate on the amorphous portion of the semicrystalline polymer [39]. Fluorescence microscopy of the modified films enriched with active phenolic agents proved no bacterial cells on the surface. This experiment confirmed that biodegradable polymer films with eugenol or thymol prevent a biofilm formation by bacterial isolates from the dairy products. These results correspond with proven antibacterial properties (see Section 3.1).

### 3.3. Material Properties 

The prepared polymer films were homogeneous and compact with a smooth surface. PLA films were clear and elastic with a slight shade of yellow. Modified PLA films were visually darker yellow due to the color of eugenol and thymol. PBAT films exhibited higher elasticity in comparison to PLA samples. Pure PBAT films were white, while eugenol and thymol also caused the change in the film to light yellow. On the other hand, all prepared PBS films were fragile with a similar yellowish color tint as PBAT films.

#### 3.3.1. Contact Angle

The wettability of polymer surfaces evidences their hydrophilicity or hydrophobicity, significantly affecting their potential applications. The wetting properties of the samples without and with active agents were analyzed by the sessile drop method. As can be seen from the values of contact angles (Table 3), prepared samples exhibited contact angles up to 90°. The base polymer films showed values ranging from 55 to 75°. More hydrophobic surfaces (103 to 120°) were observed in PBS membrane or PBS-based foam [40].

**Figure 2:**
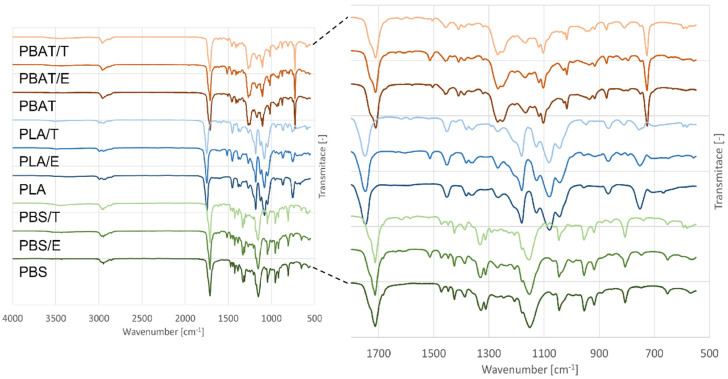
The FTIR spectra of pure and modified polymers; PLA: poly(lactic) acid, PBAT: poly(butylene adipate-co-terephthalate), PBS: poly(butylene succinate), E: 3% *w*/*v* eugenol, T: 3% *w*/*v* thymol.

The modification of PLA film with eugenol and thymol resulted in a slight increase in wettability. A rather opposite trend, an increase in hydrophobicity, was shown in PBS and PBAT samples even though the differences were not statistically significant at the p level of 0.05. In spite of this fact, ATR-FTIR analysis (Figure 2) proved the presence of phenolic groups, and the results of microbiological testing clearly revealed the antibacterial properties and biofilm reduction in modified samples (Section 3.1 and Section 3.2). No significant changes (*p* < 0.05) in wettability were observed when thymol and eugenol active agents were compared, which is probably due to the similar molecular structures of these phenolic major components of thyme and clove essential oils, respectively. A lower contact angle value in pure PBAT film could consist of its molecular structure containing more oxygen substituents with a hydrophilic character. In contrast, PLA is composed of polar and non-polar substituents in its molecular structure resulting in higher wetting angle values than those of PBAT. The incorporation of non-polar substances (thymol, eugenol) increases the overall hydrophobicity of the PBAT polymer [41]. Similarly, in the study of Moustafa et al. [42], who investigated the PBAT/coffee grounds composites for food packaging applications, a higher contact angle with the addition of torrefied coffee grounds was observed. It is worth stating that the values could be strongly affected by several factors, such as the surface roughness of the samples and the location of a droplet’s placement.

#### 3.3.2. FTIR-ATR

Infrared spectroscopy was used to characterize the main functional groups of the base polymers and reveal potential interactions between them and added active phenolic compounds. The FTIR spectra of pure and modified polymers are shown in Figure 2. The base polymers revealed the peaks corresponding to their main characteristic bonds [43,44]. Regarding PBS polymer, a very weak absorption band in the range of 3430–3500 cm^−1^ is assigned to polymer chain terminal –OH groups; -CH-groups were identified in the range of 3000–2800. Several overlapping peaks were observed in the –C=O region, relating to ester carbonyl groups of semicrystalline polyesters. In PBAT polymer spectra, the main absorption peaks at 2958 and 2869 cm^−1^ occurred correspondingly to -CH_2_ groups [43]. The peaks of PLA in the range of 3000 to 2900 cm^−1^ were assigned to –C-H vibrations of the CH_3_ side chains groups. PLA spectrum showed characteristic peaks at 1747, 2945, 2995 cm^−1^ assigned to C=O, -CH_3_ asymmetric, and -CH_3_ symmetric, respectively [44,45].

When active compounds were added, new peaks in the range from 1600 to 500 cm^−1^ were observed, indicating their interactions with polymer.

All tested polymer films showed the peaks associated with thymol presence at 1618, 1585, and 1519 cm^−1^ referring to characteristics C=C peaks corresponding to thymol aromatic ring [46,47]. The peaks about 1638, 1610, and 1514 cm^−1^ (in PLA/E film) are in suitable agreement with the characteristic structural spectrum of eugenol [48]. In a detailed analysis of IR spectra (Supplementary Materials Figure S1), a change in the form of more intensive peaks in the range from 3500 to 3400 cm^−1^ can be observed in modified polymer films. These could be assigned to phenolic groups of thymol and eugenol active agents.

## 4. Discussion

In this experiment, polymer films were prepared from synthetic polymers: PLA, PBAT, PBS. A biofilm formation was investigated on all films by various detection techniques, such as chemical (Christensen method), biological (MTT assay), and microscopic (fluorescence microscopy) [5,49]. The staining techniques belong to the least sensitive reproducible methods for determining total biofilm biomass. A safranin dye can be used for biofilm detection, as described by Ojima et al. [50] and Nguyen et al. [51]. This staining technique, similar to the Christensen method, was applied to the strain of *E. coli*. Marcos-Zambrano et al. [52] used crystal violet as in this study. Considering different biomass staining methods, it could be concluded that these staining methods are often inappropriate due to their low sensitivity [49,53]. The basic technique for visual detection of biofilm formation is light microscopy. It is a simple, inexpensive, and easy method; however, its resolution is low in comparison with other microscopic techniques [5]. Nevertheless, according to the previous studies by Azaredo et al. [5] and Hassan et al. [54], biofilm-based staining methods are one of the cheapest and most effective methods. Neither the Christensen method nor MTT assay confirmed the growth of a biofilm on PLA. Morohoshi et al. did not confirm the presence of a biofilm on PLA after two weeks of the cultivation in the seawater by DNA analysis of a biofilm [55]. On the other hand, fluorescence microscopy proved the highest sensitivity and biofilm formation. The presence of a biofilm was also confirmed on PBS and PBAT [55], as monitored in this study.

Polymer films from this study could be applied in food packaging. Substances with a potential antimicrobial activity may be added to ensure greater food safety and quality. Many compounds are used to increase antimicrobial protection in the food industry, such as essential oils or fatty acids [56,57]. Two compounds of essential oils, eugenol, and thymol were tested and proved favorable effects for preventing biofilm formation [58]. Several studies have confirmed the broad-spectrum antimicrobial activity of thymol and eugenol against bacteria and fungi [59,60,61]. Thymol incorporated in poly(lactide-co-glycolide) nanofibers proved a suitable preservative effect in wrapping strawberries in the study of Zhang et al. Therefore, thymol is beneficial to prolong the shelf life and keep the food fresh without affecting the flavor [62]. Hamzah et al. investigated various concentrations of these phenolic compounds against *Escherichia coli*, *Staphylococcus* *aureus*, *Pseudomonas aeruginosa* biofilm. The concentration of 0.96% *w*/*v* proved a high inhibition against the selected bacteria, with thymol showing the highest ability to cease a biofilm formation [63,64]. The antibacterial activity of phenolic compounds is attributed to phenolic hydroxyl in their structure [65]. Eugenol with the concentration of 0.625 µg/mL does not affect the bacterial viability; nonetheless, it downregulates the expression of virulence genes involved in the adhesion and biofilm formation (*brp*A, *com*DE, *ftf*, *gbp*B, *gtf*B, *gtf*C, *rel*A, *smu*630, *spa*P, and *vic*R) [66].

Therefore, eugenol and thymol in the concentration of 3% *w*/*v* were selected for incorporation into biodegradable polymeric films (PLA, PBAT, PBS) to prevent bacterial biofilm formation and possible occurrence of pathogenic microorganisms. These phenolic monoterpenes play an important role in the enhancement of the antimicrobial properties of polymers. They are released through the polymer matrix over time, continuously available, and diffused through the bacterial cell membrane, thereby providing the antimicrobial effect [67]. In this study, the disk diffusion method proved the antimicrobial properties of eugenol and thymol. The presence of eugenol or thymol in the tested materials was confirmed by FTIR analysis, and hydrophobic characteristics of their surface were described. The films containing eugenol showed higher antimicrobial activity than the films with thymol, similarly as published by De Morais et al. [64].

The improvement of the antimicrobial properties of PLA was achieved by the addition of thymol in the concentration of 8% *w*/*v* in the study by Ramos et al. [68]. Moderate antimicrobial activity for *Bacillus cereus* and *Staphylococcus aureus* was also detected for eugenol-grafted PLA. However, as in this work, the antimicrobial activity for *Escherichia coli* was not confirmed [69]. The advancement of the antimicrobial properties of biodegradable eugenol-grafted polymers was demonstrated in other studies. Grafted eugenol and carvacrol in chitosan nanoparticles improved the antimicrobial properties against *Escherichia coli* and *Staphylococcus aureus*, conferred antioxidant properties to the nanoparticles, and reduced cytotoxicity over pure essential oils [70]. The minimum inhibitory concentration of incorporated eugenol required for the film to exhibit antimicrobial activity was ≥40 μg/g for bacteria and ≥80 μg/g polyhydroxybutyrate (PHB) for fungi. The increase in eugenol concentration, i.e., ≥200 μg/g PHB, resulted in the complete growth inhibition of both bacteria and fungi in Narayanan et al. However, the antimicrobial activity of eugenol in the film decreased in the following order: water >3.0% acetic acid > n-hexane > 50% ethanol due to the specific migration [27]. Therefore, the concentration of 3% *w*/*v* of active substances was selected to ensure more significant antimicrobial properties of the incorporated films.

The addition of active ingredients to the polymer films has demonstrated biofilm inhibition and antimicrobial properties. Therefore, this could cause a prolongation of the biodegradation time as the enzymatic activity of the microorganisms initiates this. Pure polymers have been determined to biodegrade under various abiotic conditions. Polymer PLA is biodegradable under industrial composting conditions (58 °C, high humidity, aerobic). Polymers PBAT and PBS are biodegradable not only in composting but also in the soil (25 °C, humidity, aerobic) [71,72,73].

## 5. Conclusions

This study investigated biofilm formation on the polymer films with a perspective application in the food industry as the packaging material. PLA, PBS, and PBAT polymer films were tested for biofilm formation using various detection techniques (Christensen method, MTT assay, and fluorescence microscopy). The individual methods were compared, and their effectiveness was determined. Fluorescence microscopy proved to be the most sensitive for biofilm detection. The incorporation of the active compounds (thymol and eugenol) into the polymers mentioned above was also performed to prevent a biofilm formation by microorganisms that may affect the food quality. The antibacterial properties have been achieved by this modification, and biofilm formation was not detected on polymer films with incorporated antimicrobial compounds. These active packaging may enhance food safety and protect the consumer’s health. Consequently, a biofilm could form on the surface after releasing antimicrobial volatile compounds, which contribute to its biodegradation. Thus, future experiments will assess the release of antimicrobial compounds from the prepared polymers and the biodegradability test.

## Figures and Tables

**Table 1:** Antimicrobial activity determined by disk diffusion method (sample 5 mm in diameter).

Samples	*B. tequilensis* (mm)	*B. subtilis* (mm)	*B. pumilus* (mm)	*S. maltophilia* (mm)	*E. coli* (mm)	*S. aureus* (mm)
PLA	*	*	*	*	*	*
PLA/T	*	*	7.8 ± 1.2	12.5 ± 0.3	*	8.0 ± 0.4
PLA/E	9.5 ± 0.5	9.8 ± 0.3	7.3 ± 0.5	13.3 ± 0.3	*	9.0 ± 0.4
PBS	*	*	*	*	*	*
PBS/T	10.0 ± 0.4	9.5 ± 0.3	7.0 ± 0.4	*	10.5 ± 1.2	15.8 ± 0.5
PBS/E	11.8 ± 0.5	13.0 ± 0.7	12.3 ± 0.9	13.0 ± 0.4	15.8 ± 1.1	17.8 ± 0.5
PBAT	*	*	*	*	*	*
PBAT/T	*	7.3 ± 0.3	7.3 ± 0.3	6.3 ± 0.3	9.3 ± 0.3	10.3 ± 0.3
PBAT/E	7.8 ± 0.5	7.5 ± 0.3	9.5 ± 0.3	10.8 ± 0.3	6.3 ± 0.3	8.8 ± 0.5

PLA: poly(lactic) acid, PBAT: poly(butylene adipate-co-terephthalate), PBS: poly(butylene succinate), E: 3% *w*/*v* eugenol, T: 3% *w*/*v* thymol. * —no inhibition zone.

**Table 2:** Comparison of methods for evaluating biofilm formation for pure materials.

Materials	Methods	*B. tequilensis*	*B. subtilis*	*B. pumilus*	*S. maltophilia*	*E. coli*	*S. aureus*
PLA	MTT assay	−	−	−	−	−	−
	Christensen method	−	−	−	−	−	−
	Fluorescence microscopy (LIVE)	+++	+++	+++	+	+++	+++
	Fluorescence microscopy (DEAD)	+	−	−	+	++	++
PBS	MTT assay	+	+	+	+	+	+
	Christensen method	−	−	+	+	−	−
	Fluorescence microscopy (LIVE)	−	++	−	++	−	+
	Fluorescence microscopy (DEAD)	++	+	++	+	+++	+
PBAT	MTT assay	−	−	−	−	−	−
	Christensen method	+	+	+	+	+	+
	Fluorescence microscopy (LIVE)	+	+	+	+	+++	+
	Fluorescence microscopy (DEAD)	−	−	−	−	+++	−

PLA: poly(lactic) acid, PBAT: poly(butylene adipate-co-terephthalate), PBS: poly(butylene succinate). MTT assay and Christensen method: −: non-biofilm formation, +: with weak biofilm formation, ++: with strong biofilm formation (*p* < 0.003). Fluorescence microscopy: −: without microorganisms, +: 1–10 microorganisms, ++: 10–50 microorganisms, +++: >50 microorganisms.

**Table 3:** Contact angles values for tested polymer films.

Active Compounds	PLA (°)	PBS (°)	PBAT (°)
*	75 ± 4 ^aA^	74 ± 2 ^aA^	56 ± 4 ^aB^
3% *w*/*v* thymol	67 ± 3 ^aAB^	75 ± 4 ^aA^	60 ± 4 ^aB^
3% *w*/*v* eugenol	66 ± 2 ^aA^	74 ± 2 ^aB^	63 ± 3 ^aA^

PLA: poly(lactic) acid, PBAT: poly(butylene adipate-co-terephthalate), PBS: poly(butylene succinate), *—without active compounds. Different lower-case/upper-case letters in the same column/line indicate significant differences, respectively (*p* < 0.05).

## Data Availability

Not applicable.

## Supplementary Materials

The following are available online at https://www.mdpi.com/article/10.3390/foods11010002/s1, Figure S1: The FTIR spectra of pure and modified polymers in the range 4000 to 3000 cm^−1^; PLA: poly(lactic) acid, PBAT: poly(butylene adipate-co-terephthalate), PBS: poly(butylene succinate), E: 3% *w*/*v* eugenol, T: 3% *w*/*v* thymol., Table S1: Comparison of methods for evaluating biofilm formation.
