# Supplementary material for: Biofilm Formation Reduction by Eugenol and Thymol on Biodegradable Food Packaging Material
Source: Foods. 2021 Dec 21;11(1):2. doi: 10.3390/foods11010002 (PMC8750975; doi:10.3390/foods11010002)
Supplement: Supplementary file 1 [file foods-11-00002-s001.zip › foods-1490129-supplementary.pdf]

foods

Supplementary Materials

Biofilm formation reduction by eugenol and thymol on biodegradable food packaging material

Pavel Pleva [1], Lucie Bartošová[1] Daniela Máčalová[1], Ludmila Zálešáková[2], Jana Sedlaříková [3] and Magda Janalíková [1,*]

[1] Department of Environmental Protection Engineering, Faculty of Technology, Tomas Bata University in Zlin, 275 Vavreckova, 76001 Zlin, Czech Republic; ppleva@utb.cz
[2] Department of Food Technology, Faculty of Technology, Tomas Bata University in Zlin, nam. T. G. Masaryka 5555, 76001, Zlin, Czech Republic; e-mail@e-mail.com
[3] Department of Fat, Surfactant and Cosmetics Technology, Faculty of Technology, Tomas Bata University in Zlin, 275 Vavreckova, 76001 Zlin, Czech Republic; e-mail@e-mail.com
* Correspondence: e-mail@e-mail.com; Tel.: (optional; include country code; if there are multiple corresponding authors, add author initials)

In this Supplementary Materials, the following tables and figure are presented:

Table S1. Comparison of methods for evaluating the biofilm formation.

Figure S1. The FTIR spectra of pure and modified polymers in the range 4000 to 3000 cm^{-1}; PLA: poly(lactic) acid, PBAT: poly(butylene adipate-co-terephthalate), PBS: poly(butylene succinate), E: 3% w/v eugenol, T: 3% w/v thymol.

Table S1. Comparison of methods for evaluating the biofilm formation.

Material	Method	*B. tequilensis* R23	*B. subtilis* R25	*B. pumilus* R34	*S. maltophilia* GK CIP 1/1	*E. coli* ATCC 25922	*S. aureus* ATCC 25923
PLA	MTT assay	-	-	-	-	-	-
	Christensen method	-	-	-	-	-	-
	Fluorescence microscopy (LIVE)	+++	+++	+++	+	+++	+++
	Fluorescence microscopy (DEAD)	+	-	-	+	++	++
PLA/T	MTT assay	-	-	-	-	-	-
	Christensen method	-	-	-	-	-	-
	Fluorescence microscopy (LIVE)	-	-	-	-	-	-
	Fluorescence microscopy (DEAD)	-	-	-	-	-	-
PLA/E	MTT assay	-	-	-	-	-	-
	Christensen method	-	-	-	-	-	-
	Fluorescence microscopy (LIVE)	-	-	-	-	-	-
	Fluorescence microscopy (DEAD)	-	-	-	-	-	-
PBS	MTT assay	+	+	+	+	+	+
	Christensen method	-	-	+	+	-	-
	Fluorescence microscopy (LIVE)	-	++	-	++	-	+
	Fluorescence microscopy (DEAD)	++	+	++	+	+++	+
PBS/T	MTT assay	-	-	-	-	-	-
	Christensen method	-	-	-	-	-	-
	Fluorescence microscopy (LIVE)	-	-	-	-	-	-
	Fluorescence microscopy (DEAD)	-	-	-	-	-	-
PBS/E	MTT assay	-	-	-	-	-	-
	Christensen method	-	-	-	-	-	-
	Fluorescence microscopy (LIVE)	-	-	-	-	-	-
	Fluorescence microscopy (DEAD)	-	-	-	-	-	-
PBAT	MTT assay	-	-	-	-	-	-
	Christensen method	+	+	+	+	+	+
	Fluorescence microscopy (LIVE)	-	-	-	+	+++	-
	Fluorescence microscopy (DEAD)	-	-	-	-	+++	-
PBAT/T	MTT assay	-	-	-	-	-	-
	Christensen method	-	-	-	-	-	-
	Fluorescence microscopy (LIVE)	-	-	-	-	-	-
	Fluorescence microscopy (DEAD)	-	-	-	-	-	-
PBAT/E	MTT assay	-	-	-	-	-	-
	Christensen method	-	-	-	-	-	-
	Fluorescence microscopy (LIVE)	-	-	-	-	-	-
	Fluorescence microscopy (DEAD)	-	-	-	-	-	-

PLA: poly(lactic) acid, PBAT: poly(butylene adipate-co-terephthalate), PBS: poly(butylene succinate), T: 3% w/v thymol, E: 3% w/v eugenol.

MTT assay and Christensen method: -: non-biofilm formation, +: with weak biofilm formation, ++: with strong biofilm formation ($p < 0.003$).

Fluorescence microscopy: -: without microorganisms, +: 1–10 microorganisms, ++: 10–50 microorganisms, +++: > 50 microorganisms.

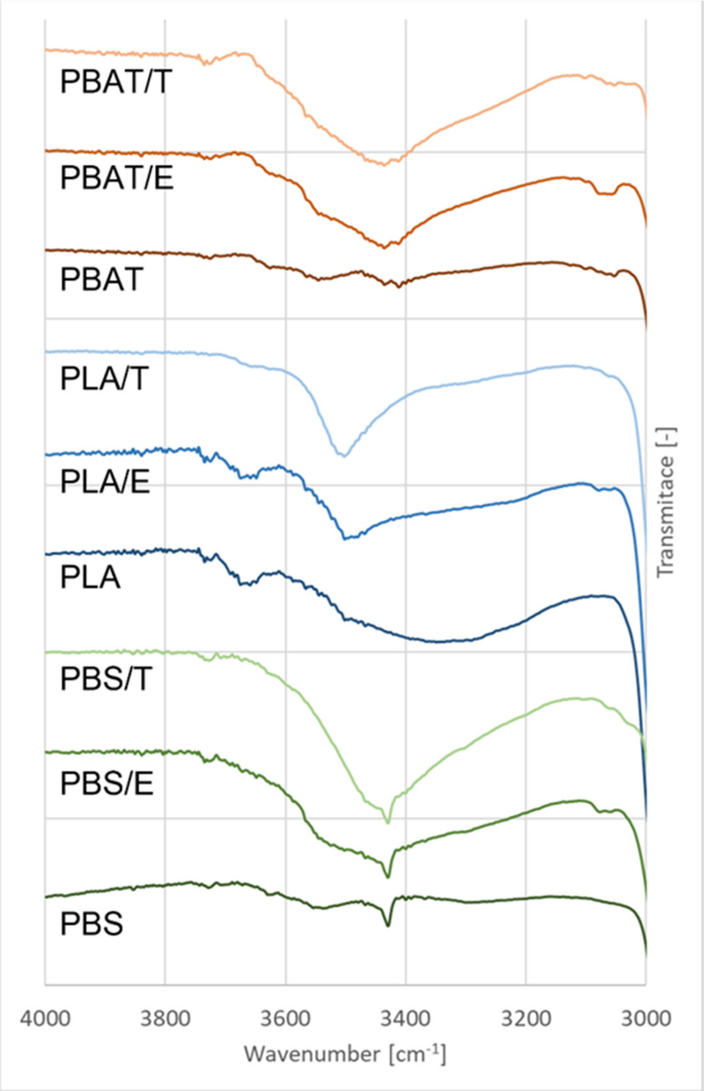

Figure S1. The FTIR spectra of pure and modified polymers in the range 4000 to 3000 cm^{-1}; PLA: poly(lactic) acid, PBAT: poly(butylene adipate-co-terephthalate), PBS: poly(butylene succinate), E: 3% w/v eugenol, T: 3% w/v thymol.
